# Long-term trends and the role of health resources in under-5 mortality rates: a 2000–2021 longitudinal analysis at the global level

**DOI:** 10.1136/bmjopen-2025-102980

**Published:** 2025-11-16

**Authors:** Zeping Zang, Lianlong Yu, Suyun Li, Xiaohui Xu, Han Zhou, Qing Yue, Li Yang

**Affiliations:** 1 School of Public Health, Cheeloo College of Medicine, Shandong University, Jinan, China; 2 Shandong Center for Disease Control and Prevention, Jinan, China; 3 School of Public Health, Shandong First Medical University & Shandong Academy of Medical Sciences, Jinan, China; 4 School of Public Health, Shandong Second Medical University, Weifang, China; 5 National Center for Chronic and Non-Communicable Disease Control and Prevention, Chinese Center for Disease Control and Prevention, Beijing, China; 6 National Center for Women and Children’s Health, NHC, PRC, Beijing, China; 7 Jinan Center for Disease Control and Prevention, affiliated with Shandong University, Jinan, China

**Keywords:** Health policy, Child, PUBLIC HEALTH, Health Services

## Abstract

**Abstract:**

**Objective:**

The under-5 mortality rate (U5MR) is a crucial global health metric for evaluating public health interventions, and further reductions in U5MR are essential for achieving the Sustainable Development Goals (SDGs). This study analyses the trends of U5MR globally from 2000 to 2021 and explores the quantitative impact of health resources on U5MR reduction.

**Design and participants:**

This study used WHO public data from 2000 to 2021 to investigate the temporal trend of U5MR through Joinpoint regression analysis. A two-way fixed-effect model was used to investigate the relationship between U5MR and health resources.

**Primary outcome measures:**

Data on U5MR and health resources (including six vaccine-related indicators and eight health expenditure-related indicators) were obtained from the WHO Global Health Observatory, encompassing 200 countries and regions from 2000 to 2021.

**Results:**

Globally, U5MR declined, though at a slower pace (annual average percentage change: −3.259, p<0.001), while vaccination coverage and health expenditures increased (p<0.05). We found a significant negative correlation of global polio vaccination coverage (β=−0.489, p<0.05) and current health expenditure (CHE) as percentage of gross domestic product and U5MR (β=−0.762, p<0.05) with U5MR. In G20 countries, domestic general government health expenditure as percentage of CHE was negatively correlated with U5MR (β=−0.553, p<0.05). Health resources contributed to 65.01% of U5MR reduction in G20 countries, with vaccines accounting for 23.86%. Globally, health resources contributed 37.26% to U5MR reduction, with vaccines accounting for 72.69%.

**Conclusion:**

Global U5MR has declined from 2000 to 2021, but progress remains insufficient to fully achieve the SDGs. Immunisation played a dominant role in the global reduction of U5MR, underscoring the critical need to prioritise vaccination in health resource allocation strategies.

**STRENGTHS AND LIMITATIONS OF THIS STUDY**

- The study leverages a comprehensive WHO dataset spanning 200 countries and regions (2000–2021) for the analysis of under-5 mortality rate (U5MR) trends.
- A two-way fixed effects model was employed to control for unobserved country heterogeneity and common temporal shocks, strengthening the basis for causal inference.
- The dominance analysis quantitatively assesses the impact of key health resource investments (vaccines and expenditures) on reducing U5MR.
- A limitation is the lack of stratified variables (eg, age, gender), which precluded examination of their influence on the outcomes.
- Additionally, the absence of data on individual vaccine doses prevented assessment of how immunisation completeness affects U5MR.

## Introduction

 In recent years, substantial advancements have been made globally in improving child health. Notably, between 2000 and 2015, Millennium Development Goal 4 (MDG 4) explicitly set an ambitious objective to decrease under-5 mortality rate (U5MR) by two-thirds compared with the 1990 level.^1^ From 2000 to 2013, the annual number of under-5 deaths declined from 12.7 million to 6.3 million. Concurrently, the U5MR dropped by 49%, from 90 to 46 per 1000 live births. This period marked an unprecedented acceleration in the reduction of child mortality, with 58 countries successfully attaining the MDG 4 target.^1 2^

The challenge of improving global child health remains, as evidenced by the current global U5MR of 36.7 per 1000 live births, which is still significantly above the target established by the Sustainable Development Goals (SDGs). The SDGs call for a reduction in U5MR to a maximum of 25 deaths per 1000 live births worldwide by 2030.^3^ Of the 200 countries and regions analysed, 146 have achieved or are on track to achieve the specific SDGs target for reducing U5MR. Despite this progress, numerous countries continue to face substantial challenges in this regard.

The increasing investment in health resources during the MDG 4 period is widely considered a significant role in reducing U5MR. A global comparative analysis found that health expenditure can reduce U5MR, especially in low-income countries.^4^ Another study in Africa found that countries with increased vaccine immunisation have achieved greater reductions in U5MR.^5^ The impact of vaccination is substantial at a global scale, being estimated to prevent 21.7% of deaths in children under 5 years old.^6^ The case of polio illustrates this point: studies in Guinea-Bissau and Bangladesh showed that oral polio vaccine was associated with a reduction in child mortality by 19%–31%.^7 8^

To achieve SDGs, it is imperative to understand how the effect of health resource investments on U5MR has changed from 2000 to 2021, particularly the role of vaccination. Therefore, this study aims to analyse the 22-year trends in U5MR from 2000 to 2021 and to quantify the influence of health resource investments, especially vaccination.

## Methods

### Data sources

The data for this study were sourced from the WHO Global Health Observatory (https://www.who.int/data/gho, accessed on 12 September 2024) (dataset).^9^ The primary assessment variables, spanning the period from 2000 to 2021, cover 200 countries and regions and six major geographical regions. We analysed U5MR, defined as the number of deaths of children under 5 years old per 1000 live births, in relation to various health resource indicators. The health resources indicators included in the analysis were hepatitis B immunisation coverage among 1-year-olds (HepB3, %), neonates protected at birth against neonatal tetanus (PAB, %), Haemophilus influenzae type b immunisation coverage among 1-year-olds (Hib3, %), BCG immunisation coverage among 1-year-olds (BCG, %), polio immunisation coverage among 1-year-olds (Pol3, %), measles-containing-vaccine first-dose immunisation coverage among 1-year-olds (MCV1, %), domestic general government health expenditure as percentage of general government expenditure (GGE) (GGHE-D,%), current health expenditure (CHE) per capita in US$, CHE as percentage of gross domestic product (GDP) (%), external health expenditure (EXT) per capita in US$, EXT as percentage of CHE (%), GGHE-D per capita in US$, GGHE-D as percentage of CHE (%) and GGHE-D as percentage of GDP (%).

### Statistical analyses

The descriptive analysis was conducted to summarise the U5MR, immunisation coverage for six vaccines, CHE as a percentage of GDP, and GGHE-D as a percentage of CHE at the global level, across six major regions, and among the G20 countries. The long-term trends in U5MR were assessed using the best-fitting segmented Joinpoint continuous log-linear model, with the annual average percentage change (AAPC) calculated.

Countries with severe missing data were excluded from subsequent analyses. The Hausman test was employed to guide the choice between using the fixed effect model or the random effect model. Additionally, the joint significance test was used to determine the inclusion of time fixed effect. A two-way fixed effects model with cluster-robust standard errors, clustered by country, was employed to examine the relationships between U5MR and key health resource indicators, including vaccine immunisation coverage and health expenditures. These analyses were conducted for 139 countries globally and for G20 countries specifically, spanning the period from 2000 to 2021. The contribution of each variable to the model was assessed using dominance analysis, which estimates the relative importance of independent variables. The variables with a variance inflation factor >10 were excluded by the multi-collinearity test. The independent variables included coverage rates for HeB3, Hib3, Pol3, GGHE-D as percentage of GGE, CHE per capita in $, CHE as percentage of GDP and GGHE-D as percentage of CHE. Statistical analysis was performed using Stata V.18.0, ArcMap V.10.8 and Joinpoint V.5.0. A p value of <0.05 (two-tailed) was considered statistically significant.

## Results

**Figure 1:**
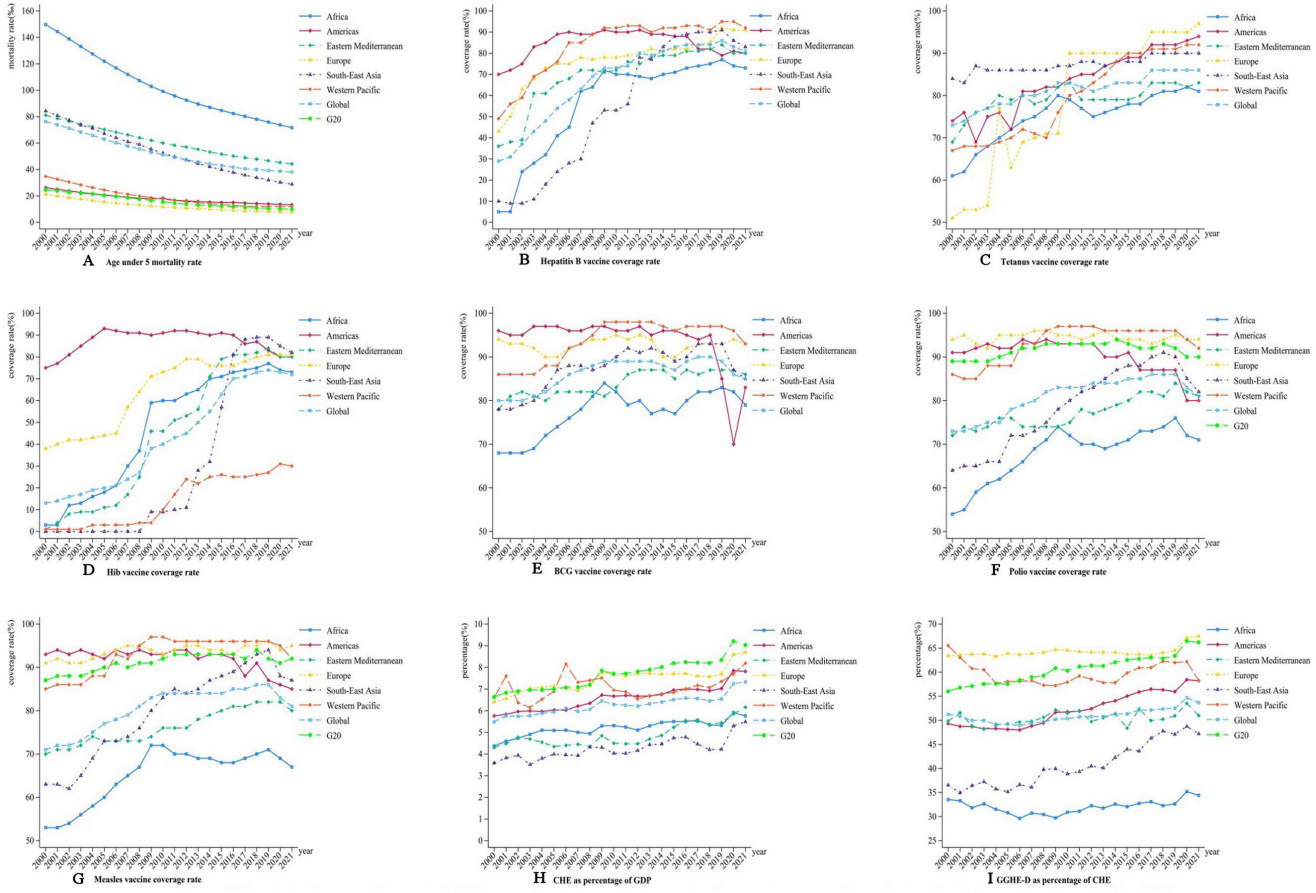
Trends in the mortality rate of children under 5 years, the immunisation coverage rate of vaccine and health spending from 2000 to 2021. CHE, current health expenditure; GDP, gross domestic product; GGHE-D, domestic general government health expenditure.

Tables1  2 summarise the descriptive statistics for U5MR and health resources in G20 countries and six major regions at three time points: 2000, 2010 and 2021. U5MR in both G20 countries and the six major regions was significantly lower in 2021 compared with 2000 and 2010, reflecting a notable decline since 2000. The immunisation coverage for polio maintained an overall upward trend, with the exception of a few countries and the Americas. Both the proportion of CHE relative to GDP and the share of GGHE in total health expenditure increased relative to previous years. These trends are further depicted in figure 1.

**Table 2:** Basic information on vaccine coverage in G20 countries and six regions

	Hepatitis B vaccine coverage (％)			Hib vaccine coverage (％)			Polio vaccine coverage (％)			Measles vaccine coverage (％)		
	2000	2010	2021	2000	2010	2021	2000	2010	2021	2000	2010	2021
G20 countries												
Argentina	–	94	81	83	94	81	88	95	79	91	95	86
Australia	–	92	95	90	92	95	90	92	95	91	94	93
Brazil	94	96	68	90	99	68	99	99	68	99	99	73
Canada	–	56	84	88	90	92	88	90	92	96	90	90
China	60	99	99	–	–	–	86	99	99	84	99	99
Germany	84	88	87	94	93	90	94	94	91	92	97	97
French	26	64	95	86	97	96	98	99	96	84	89	94
UK	–	–	93	91	94	93	91	94	93	88	89	91
Indonesia	65	83	67	–	–	67	72	82	68	76	78	72
India	–	38	85	–	–	85	57	76	85	56	82	89
Italy	94	96	94	55	95	94	97	96	94	74	91	94
Japan	–	–	96	–	–	99	98	98	99	96	94	95
Korea	93	94	98	–	–	98	99	95	98	95	98	98
Mexico	97	93	80	97	95	78	97	95	78	96	95	99
Russia	–	97	97	–	–	–	97	98	97	97	98	97
Saudi Arabia	93	98	97	–	98	97	95	98	97	94	98	98
Türkiye	71	96	96	–	97	95	85	97	95	87	97	96
America	90	92	92	93	90	91	90	93	93	91	92	92
South Africa	73	71	86	73	72	86	71	72	86	72	72	87
Region												
Africa	5	70	73	3	60	73	54	72	71	53	72	67
Americas	70	90	80	75	91	80	91	93	80	93	93	85
Eastern Mediterranean	36	72	80	1	46	80	72	75	81	70	76	80
Europe	43	78	91	38	73	81	94	95	94	91	93	95
South-East Asia	10	53	83	0	9	82	64	80	82	63	83	87
Western Pacific	49	92	92	1	10	30	86	97	92	85	97	92

**Table 1:** Basic information on under-5 mortality and health spending in G20 countries and six regions

	U5MR per 1000 live births			CHE as percentage of GDP (%)			GGHE-D as percentage of CHE (%)		
	2000	2010	2021	2000	2010	2021	2000	2010	2021
G20 countries									
Argentina	19.37	14.47	9.72	8.22	9.45	9.71	54.73	58.95	63.21
Australia	6.19	4.77	3.77	7.59	8.42	10.54	71.84	72.36	76.04
Brazil	34.63	18.63	14.28	8.33	7.95	9.89	41.64	45.02	45.54
Canada	6.23	5.73	5.02	8.25	10.68	12.33	72.85	69.88	72.92
China	36.72	15.74	7.03	4.51	4.23	5.38	21.98	51.91	54.07
Germany	5.36	4.18	3.62	9.89	11.10	12.93	78.20	75.69	79.05
French	5.39	4.21	4.13	9.58	11.23	12.31	72.73	70.44	75.62
UK	6.55	5.17	4.18	7.13	9.93	12.36	77.08	81.09	83.69
Indonesia	52.05	33.68	22.01	1.85	2.79	3.71	29.67	23.69	59.41
India	91.68	58.12	30.60	4.03	3.27	3.28	20.68	26.21	34.27
Italy	5.57	3.96	2.75	7.57	8.92	9.38	72.64	78.45	75.46
Japan	4.51	3.20	2.34	7.03	9.06	10.82	80.43	81.93	84.72
Korea	7.54	4.13	2.89	3.86	5.82	9.33	50.61	58.40	61.00
Mexico	28.16	19.15	13.28	4.45	5.74	6.08	45.22	50.22	50.10
Russia	19.37	10.41	5.05	5.02	4.97	7.39	59.36	61.38	71.17
Saudi Arabia	22.07	12.24	6.69	4.21	3.65	5.97	72.05	61.93	76.98
Türkiye	37.59	17.91	10.06	4.60	5.02	4.57	61.68	78.00	78.80
America	8.45	7.34	6.33	12.49	16.20	17.36	44.38	48.85	55.40
South Africa	71.02	51.64	34.70	7.34	7.79	8.27	36.59	51.33	60.38
Region									
Africa	149.75	99.16	71.67	4.37	5.31	5.76	33.52	30.90	34.41
Americas	26.24	18.26	13.25	5.76	6.67	7.81	49.33	51.74	58.21
Eastern Mediterranean	81.14	60.06	44.17	4.30	4.50	6.16	49.85	51.49	50.98
Europe	21.13	11.77	7.65	6.40	7.69	8.70	63.39	64.55	67.45
South-East Asia	84.26	52.48	28.85	3.58	4.03	5.49	36.53	38.85	47.19
Western Pacific	34.80	17.60	11.92	6.60	6.95	8.19	65.53	57.92	58.25

CHE, current health expenditure; GDP, gross domestic product; GGHE-D, domestic general government health expenditure; U5MR, under-5 mortality rate.

**Figure 2:**
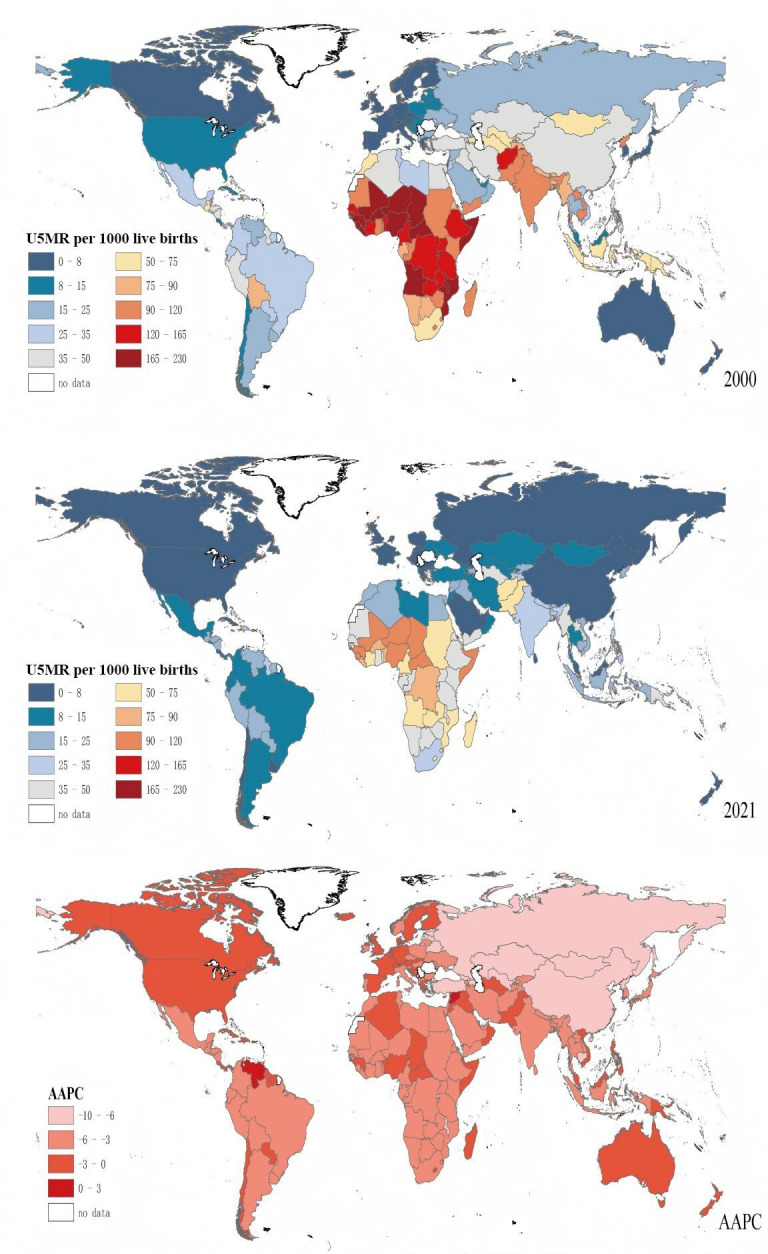
Maps of global age under-5 mortality rate for 200 countries and regions. AAPC, annual average percentage change; U5MR, under-5 mortality rate.

Figure 2 presents the changes in U5MR across 200 countries and regions in 2000, 2010 and 2021. U5MR has shown a rapid decline globally, with the most notable reductions observed in Africa. However, U5MR remains high in certain regions of Africa, South Asia and South-East Asia.

Figure 1 presents a line chart illustrating the trends in U5MR, vaccination coverage and health expenditures across 200 countries and regions, categorised by region. As shown in figure 1B–G, the immunisation coverage for the six vaccines demonstrates a significant upward trend (p<0.05).

Figure 1A illustrates a decline in U5MR globally, across six major regions, and in G20 countries (AAPC: −5.188 to −2.749, p<0.05), although the rate of decline has decelerated over time. U5MR still remains notably high in the African, Eastern Mediterranean and Southeast Asian regions, with Africa and the Eastern Mediterranean exhibiting rates above the global average.

Figure 1B demonstrates an increase in hepatitis B vaccination coverage (AAPC: 0.1748 to 18.0608, p<0.05), although coverage in Africa, the Eastern Mediterranean and the Americas remains below the global average.

Figure 1C illustrates an increase in tetanus vaccine coverage (AAPC: 0.2504 to 4.1797, p<0.05), though coverage in Africa and the Eastern Mediterranean continues to lag behind the global average.

Figure 1D depicts an increase in Hib vaccine coverage (AAPC: 0.0239 to 29.0832, p<0.05), with the Western Pacific region showing substantially lower coverage compared with the global average.

Figure 1E illustrates an increase in BCG vaccination coverage in Africa and the Western Pacific (AAPC: 0.1103 to 1.3561, p<0.05), while a decline is observed in the Americas (AAPC: −1.1091 to −0.2174, p<0.05), where coverage has rapidly decreased in recent years. Currently, vaccination coverage in both Africa and the Americas remains below the global average.

Figure 1F presents an increase in polio vaccination coverage in Africa, the Eastern Mediterranean, Southeast Asia and the Western Pacific (AAPC: 0.0298 to 1.8576, p<0.05), whereas coverage is declining in the Americas (AAPC: −0.8071 to −0.3318, p<0.05). Polio vaccination coverage remains relatively low in Africa, the Eastern Mediterranean and Southeast Asia, with Africa and the Americas showing coverage rates below the global average.

Figure 1G highlights an increase in measles vaccination coverage (AAPC: 0.0317 to 2.1917, p<0.05), with a declining trend in the Americas (AAPC: −0.6277 to −0.2845, p<0.005). Coverage remains below the global average in Africa and the Eastern Mediterranean.

Figure 1H demonstrates an upward trend in the proportion of CHE as a share of GDP in Africa, the Americas, Europe, South-East Asia, globally, and in G20 countries (AAPC: 0.6642 to 3.3489, p<0.05). However, CHE as a percentage of GDP remains consistently lower in Africa, the Eastern Mediterranean and South-East Asia compared with the global average. Figure 1I highlights an increasing trend in GGHE as a percentage of CHE (GGHE-D/CHE) in the Americas, Eastern Mediterranean, Europe, South-East Asia, globally and G20 countries (AAPC: 0.0098 to 1.9016, p<0.05). In contrast, the Western Pacific region exhibits a declining trend (AAPC: −0.9077 to −0.0674, p<0.05). Africa, the Eastern Mediterranean and South-East Asia continue to fall below the global average, with Africa showing a particularly pronounced lag.

**Figure 3:**
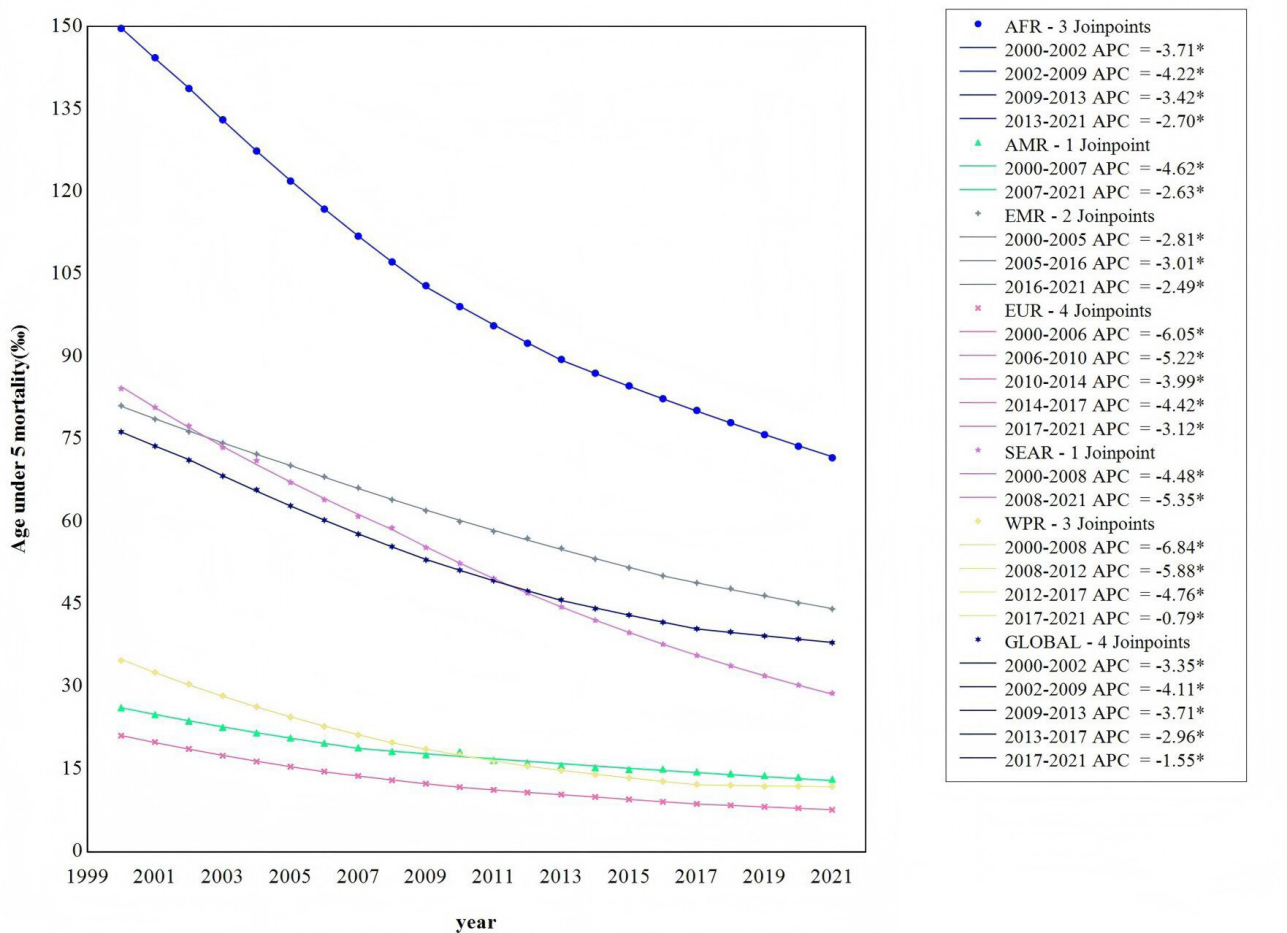
Temporal trend of age under-5 mortality rate described by Joinpoint model from 2000 to 2021. AFR, African region; AMR, region of the America; APC, annual average percentage; EMR, Eastern Mediterranean region; EUR, European region; SEAR, South-East Asia region; WPR, Western Pacific region.

As illustrated in figure 3, the Joinpoint regression model results show a significant decline in U5MR both globally and across the regions of Africa, the Americas, the Eastern Mediterranean, Europe, South-East Asia and the Western Pacific (p<0.001). The global decline in U5MR is segmented into five phases, with Africa showing four phases, the Americas two phases, the Eastern Mediterranean three phases, Europe five phases, South-East Asia two phases and the Western Pacific five phases. The AAPC values for all regions are negative, indicating a consistent decline in U5MR from 2000 to 2021. Notably, the Eastern Mediterranean region shows the slowest rate of decline in U5MR. This is evidenced by the following AAPC values: −3.259% (95% CI −3.396% to −3.121%) globally, −3.438% (95% CI −3.461% to −3.415%) in Africa, −3.294% (95% CI −3.570% to −3.016%) in the Americas, −2.841% (95% CI −2.932% to −2.749%) in the Eastern Mediterranean, −4.715% (95% CI −4.902% to −4.528%) in Europe, −5.019% (95% CI −5.075% to −4.963%) in South-East Asia and −5.033% (95% CI −5.188% to −4.878%) in the Western Pacific region.

The R^2^ values for global vaccine immunisation coverage and health expenditure within the model were 0.1520 and 0.0571, respectively. Dominance analysis reveals that their respective contributions to the model’s R^2^ are 27.08% and 10.18%, with vaccines and health expenditure accounting for 72.69% and 27.31% of total health resources, respectively. In G20 countries, the R^2^ values for vaccine immunisation coverage and health expenditure were 0.1167 and 0.3725, respectively, and their contributions to the model’s R^2^ were 15.50% and 49.51%, with vaccines and health expenditure accounting for 23.86% and 76.14% of total health resources, respectively. The analysis reveals a statistically significant negative correlation between global pol3 coverage and U5MR (β=−0.489, p<0.05), as well as between CHE as a percentage of GDP and U5MR (β=−0.762, p<0.05). A positive correlation was observed between CHE per capita in $ and U5MR (β=0.010, p<0.05). In G20 countries, there was a negative correlation between GGHE-D as percentage CHE and U5MR (β=−0.553, p<0.05). Additionally, the positive correlation was again observed between CHE per capita in $ and U5MR (β=0.003, p<0.05) (detailed data are provided in online supplemental table 1).

## Discussion

Our study found that U5MR has been declining continuously from 2000 to 2021 globally and across 200 countries and regions. In the G20 countries, vaccine immunisation coverage and health expenditure accounted for 65.01% of the observed reduction in U5MR, with vaccines contributing 23.86% of the health resources that contributed to this reduction. At the global level, health resources contributed 37.26% to the reduction of U5MR, with vaccines representing 72.69% of the health resources within this contribution. Since 2000, the global U5MR has decreased by 50%, corresponding to an average annual percentage change of −3.259%. Previous research indicated that this decline since 2000 was associated with global investments and targeted interventions.^10 11^ Increasing investment in health resources can significantly decrease U5MR. Therefore, it is imperative for countries and the international community to sustain and expand their efforts in delivering fundamental interventions around the dual pillars of vaccine access and health expenditure.

Our study also found that higher levels of health expenditure contribute to the reduction of U5MR. This is consistent with the findings of Moreno-Serra and Smith.^12^ A 1% decrease in government health expenditure is associated with a significant increase in U5MR (R=0.5207, p<0.0001, 95% CI 0.3168 to 0.7247).^13^ Similarly, in European Union countries, a decrease in health expenditure as a percentage of GDP is significantly associated with an increase in U5MR (R=−0.5624, p=0.0000, 95% CI −0.7159 to −0.4089).^14^ In G20 countries, we found that GGHE-D as a percentage of CHE is negatively correlated with U5MR and accounted for 31.48% of the reduction in U5MR. Additionally, an increase in U5MR stimulates increased investments in health resources. Our study found a positive correlation between CHE per capita in $ and U5MR in both global level and G20 countries. This result is consistent with a previous study in West African countries.^15^ One explanation could be that rising U5MR may prompt a reactive increase in per capita health expenditure. Furthermore, inequities in service supply, potentially influenced by the mix of public and private healthcare delivery models, may also contribute to this positive relationship between health spending and U5MR. It may also be related to the inefficiency in the allocation of resources and governance issues such as corruption within health systems.^16^

The implementation and expansion of vaccination interventions are associated with reductions in U5MR. Our analysis identified a negative correlation between polio vaccination coverage and U5MR, accounting for 6.45% of global reduction in U5MR. Currently, childhood immunisation is fundamental to the eradication of polio, and increasing polio vaccination coverage is crucial for ensuring global polio eradication.^17^,^19^ Similar findings from several African and South Asian countries have shown that higher polio vaccination coverage is effective in eradicating polio.^78 20^,^23^

Vaccination is a critical factor in reducing U5MR. Dominance analysis revealed that vaccination accounts for 72.69% of health resources investments in reducing global U5MR. These findings underscore its core position in improving child survival. In G20 countries, vaccination contributes 23.86% of health resources investments. Although this contribution percentage is lower than the global level, it still demonstrates the significant value of vaccination in both developed and developing nations. Our findings indicated that vaccination remains an indispensable factor for child health, even in countries with more abundant healthcare resources.

External uncertainties, such as wars and conflicts, exacerbate the risk of death for children under 5 years of age. The global childhood vaccination coverage has decreased, thereby increasing the susceptibility of millions of children to vaccine-preventable diseases.^24^,^26^ We particularly note that global polio vaccination coverage declined since the onset of the COVID-19 pandemic in 2019.^27 28^ This decline has been especially pronounced in certain countries within the Americas, Africa and the Eastern Mediterranean region in recent years.^29^,^31^ Vaccination hesitancy may significantly contribute to the plateau or even decline in vaccination coverage. Furthermore, the effects of conflicts and wars on children warrant significant attention. The Eastern Mediterranean region, which is plagued by frequent wars, exhibits the slowest reduction in U5MR. Providing vaccines and immunisations for children remains a major challenge in eradicating polio.^32^,^35^ Wars and conflicts often lead to the collapse of local healthcare systems, preventing the provision of basic medical services, which significantly worsens health conditions and greatly increases the risk of child mortality.^36^,^39^

## Conclusion

From 2000 to 2021, U5MR declined globally, although the rate of decline decelerated over time. Overall, vaccine immunisation coverage exhibited an upward trend. Global health resources accounted for 37.26% to the reduction of U5MR, with vaccination contributing 72.69% of the impact attributed to health resources. In G20 countries, health resources accounted for a greater share (65.01%) of the reduction, with vaccination contributing 23.86%. Increasing investments in vaccine immunisation coverage and health expenditure are critical to further reduce U5MR.

## Supplementary material

10.1136/bmjopen-2025-102980 online supplemental file 1

## Data Availability

Data are available in a public, open access repository.
