# Supplementary material for: Long-term trends and the role of health resources in under-5 mortality rates: a 2000–2021 longitudinal analysis at the global level
Source: BMJ Open. 2025 Nov 16;15(11):e102980. doi: 10.1136/bmjopen-2025-102980 (PMC12625828; doi:10.1136/bmjopen-2025-102980)
Supplement: online supplemental file 1 [file bmjopen-15-11-s001.docx]

| Supplement table 1 The relationship between independent valuables and mortality of children under five | | | | | |
| --- | --- | --- | --- | --- | --- |
|  | Two-way fixed-effect model | | | | |
|  | β | 95%CI | R^2^ | Dominance analysis | Percentage of health resources |
| **Global** |  |  |  |  |  |
| *Vaccine* |  |  | 0.1520 | 0.2708 | 0.7269 |
| Hepatitis B | 0.038 | (-0.047,0.124) | 0.0235 | 0.0418 | 0.1124 |
| Hib | 0.051 | (-0.035,0.137) | 0.0245 | 0.0437 | 0.1172 |
| Polio | -0.489 | (-0.765,-0.214) | 0.0645 | 0.1149 | 0.3085 |
| Measles | -0.147 | (-0.398,0.104) | 0.0395 | 0.0704 | 0.1889 |
| *Health spending* |  |  | 0.0571 | 0.1018 | 0.2731 |
| GGHE-D as percentage of GGE | 0.080 | (-0.498,0.658) | 0.0016 | 0.0028 | 0.0077 |
| CHE per capita in $ | 0.010 | (0.007,0.013) | 0.0372 | 0.0663 | 0.1779 |
| CHE as percentage of GDP | -0.762 | (-1.494,-0.029) | 0.0166 | 0.0296 | 0.0794 |
| GGHE-D as percentage of CHE | 0.068 | (-0.113,0.249) | 0.0017 | 0.0031 | 0.0081 |
| **G20 countries** |  |  |  |  |  |
| *Vaccine* |  |  | 0.1167 | 0.1550 | 0.2386 |
| Hepatitis B | 0.060 | (-0.046,0.165) | 0.0099 | 0.0131 | 0.0202 |
| Hib | 0.254 | (-0.107,0.615) | 0.0151 | 0.0201 | 0.0309 |
| Polio | -0.264 | (-0.535,0.008) | 0.0155 | 0.0205 | 0.0317 |
| Measles | -0.318 | (-0.979,0.343) | 0.0762 | 0.1013 | 0.1558 |
| *Health spending* |  |  | 0.3725 | 0.4951 | 0.7614 |
| GGHE-D as percentage of GGE | -0.528 | (-1.396,0.340) | 0.0504 | 0.0670 | 0.1030 |
| CHE per capita in $ | 0.003 | (0.002,0.005) | 0.0531 | 0.0706 | 0.1085 |
| CHE as percentage of GDP | 0.361 | (-1.853,2.574) | 0.0322 | 0.0427 | 0.0658 |
| GGHE-D as percentage of CHE | -0.553 | (-0.991,-0.114) | 0.2368 | 0.3148 | 0.4841 |

GGHE-D: domestic general government health expenditure; GGE: general government expenditure; CHE: current health expenditure. Due to missing data, only Argentina, Australia, Brazil, Canada, Germany, France, Italy, Mexico, Saudi Arabia, Türkiye, United States and South Africa were included. Health resources here refers to the vaccine plus health spending in the table.
