# Supplementary material for: Long-term trends and the role of health resources in under-5 mortality rates: a 2000–2021 longitudinal analysis at the global level
Source: BMJ Open. 2025 Nov 16;15(11):e102980. doi: 10.1136/bmjopen-2025-102980 (PMC12625828; doi:10.1136/bmjopen-2025-102980)
Supplement: Supplementary file 2 [file bmjopen-2025-102980.reviewer_comments.pdf]

PEER REVIEW HISTORY

BMJ Open publishes all reviews undertaken for accepted manuscripts. Reviewers are asked to complete a checklist review form and are provided with free text boxes to elaborate on their assessment. These free text comments are reproduced below.

ARTICLE DETAILS

Title (Provisional)

Long-term Trends in Under-Five Mortality Rates and the Role of Health Resources: A 2000-2021 Longitudinal Analysis at the Global Level

Authors

Zang, Zeping; Yu, Lianlong; Li, Suyun; Xu, Xiaohui; Zhou, Han; Yue, Qing; Yang, Li

---

VERSION 1 - REVIEW

**Reviewer**	**1**
**Name**	**Fraser, James**
**Affiliation**	**Bristol Royal Hospital for Children, Paediatric Intensive Care**
**Date**	**22-May-2025**
**COI**	**None**

This is generally a well written paper on an important subject. Its strengths are its 'broad scope and extensive temporal span'. The results are generally well presented although some of the key statistical 'take-ways' would benefit from further clarity and explanation. I would like to caveat my answer to q. 11 in the review checklist as 'probably' but believe confirmation of this by a statistician would be helpful.

My more detailed comments are below:

Major

Title:

'Long-term Trends in Under-Five Mortality Rates and the Role of Health Resources: A 2000-2021 Longitudinal Analysis in Global'. Poor English. Global is an adjective. Global what?

Abstract:

Results and the 'what this study adds' sections seem to contradict themselves. In the results the authors state 'Health resources contributed to 65.01% of U5MR reduction in G20 countries, with vaccines accounting for 23.86%'. In the 'What this study adds' section the

authors state differently 'In G20 countries, vaccines account for 15.50% of the reduction in U5MR, and health expenditures account for 49.51%'. Please clarify.

Introduction:

I think it would be helpful to have a glossary defining the various health expenditure indices.

Line 2 page 6 mentions 139 countries, but 200 countries mentioned in results?

Results:

Table 1, Figure 1: I think the column title 'Age under 5 mortality(‰)' is confusing. The reader might find it more straightforward if the heading here simply read 'U5MR per 1000 live births' ?

Page 6 line 15.: The authors might be more circumspect in relation to their comments about polio vaccination which actually decreased in 7 countries and the Americas as a whole during the period under study.

Figure 2 (a-g). Very clearly explained in text.

Joinpoint regression model analysis and R2 interpretation is beyond my level of statistical expertise.

Discussion:

Slightly 'wordy'. Could be shortened.

Page 9, Para1: Please advise how the figures 65% and 37.26% are arrived at?

Page 11, para 3. The finding that different measures of investment in health resources are correlated with U5MR is interesting. Why do the authors think that GGHE-D is negatively correlated whereas CHE per capita is positively correlated?

Page 11, line 15. The model of health care systems (eg private versus public) in relation to equity may also part explain the relationship between health spend and U5MR.

Para 6. The authors are right to speculate as to why global vaccination rates have plateaued and / or decreased in recent years. They might mention vaccination hesitancy in this context.

Minor:

'What this study adds' section line14: 'have significantly impact U5MR'. Poor English

Introduction:

Line 16: repeats sentence 'Domestic general government health expenditure (GGHE-D) as percentage of current health expenditure (CHE)'

Results:

Page 7 line 27: '..resources'. Might add 'respectively' for clarity.

Page 8 line 2: '..resources'. Might add 'respectively' for clarity.

Discussion:

Page 11, line 2: ' an indispensable 'what''?

Reviewer **2**

Name **Otieku, Evans**

Affiliation **University of Ghana, Institute of Statistical, Social and Economic Research**

Date **26-Jun-2025**

COI **None**

This paper is exciting to read and recognizes the need to dramatically reduce under-five mortality. While making a case for what is known about the study, the authors mentioned 49% reduction in U5MR between 2000-2013, Could they update such information with a more recent estimate of 51% reduction between 2000 and 2022 (see Murphy 2024; UNIGCME 2023). Could they check to see what has been done by the United Nations Inter-Agency Group for Child Mortality Estimate (UNIGCME) to see whether the overarching purpose of this study has not been worked on already.

It is good to point out that 146 countries are on track to achieve SDG target of 25/1000 by 2030. Should this not inform where to focus the analysis to understand why the remaining countries are lagging behind. Why the specific focus on G20 countries? Could they expand a bit more about how they addressed heterogeneity in this comparative analysis? Could they expand the reasons for the log-linear model. A better description of the statistical analysis will help, especially with regards to justification and all the underlying assumptions considered in model development.

VERSION 1 - AUTHOR RESPONSE

Reviewer: 1
Dr. James Fraser, Bristol Royal Hospital for Children
Comments to the Author:

1. *Title:*
'Long-term Trends in Under-Five Mortality Rates and the Role of Health Resources: A 2000-2021 Longitudinal Analysis in Global'. Poor English. Global is an adjective. Global what?

Answer: Thank you for pointing out the error on the title, we apologize for the poor English. This is indeed a grammatical error, and the title has been changed to "Long-term Trends in Under-Five Mortality Rates and the Role of Health Resources: A 2000-2021 Longitudinal Analysis at the Global Level". (Main Document: page 1, line 2)

2. *Abstract:*
Results and the 'what this study adds' sections seem to contradict themselves. In the results the authors state 'Health resources contributed to 65.01% of U5MR reduction in G20 countries, with vaccines accounting for 23.86%'. In the 'What this study adds' section the authors state differently 'In G20

countries, vaccines account for 15.50% of the reduction in U5MR, and health expenditures account for 49.51%'. Please clarify.

Answer: Thank you for your question. This is actually a question of total and point. First, 65.01% refers to the contribution of health resources (including vaccines and health spending) to the reduction of U5MR in G20 countries, while 23.86% refers to the proportion of vaccines to the reduction of U5MR in health resources (65.01%). Then, 15.50% and 49.51% refer to the contribution of vaccine and spending to reducing U5MR in G20 countries, respectively, and the sum of the two is actually equal to 65.01%. (See supplement table 1)

3. *Introduction:*
I think it would be helpful to have a glossary defining the various health expenditure indices. Line 2 page 6 mentions 139 countries, but 200 countries mentioned in results?

Answer: Thanks for the suggestion. We have added a glossary on health spending indices to the text (Page 2, line 10). Regarding the number of countries, 200 countries and regions were included in the variables of U5MR, but only 139 countries were included in the two-way fixed effects model due to the lack of data on vaccines and health spending due to the large year span.

4. *Results:*
Table 1, Figure 1: I think the column title 'Age under 5 mortality(‰)' is confusing. The reader might find it more straightforward if the heading here simply read 'U5MR per 1000 live births' ?

Answer: Thanks for the suggestion on title ‘Age under 5 mortality(‰)’ of table 1 and figure 1. We have changed the title to U5MR per 1000 live births. （Table 1 and figure 1）

5. Page 6 line 15.: The authors might be more circumspect in relation to their comments about polio vaccination which actually decreased in 7 countries and the Americas as a whole during the period under study.

Answer: Thank you for your suggestion. We have revised the relevant expression to 'The immunization coverage for polio maintained an overall upward trend, with the exception of a few countries and the Americas'. (Page 5, line 18)

6. Figure 2 (a-g). Very clearly explained in text.
Joinpoint regression model analysis and R2 interpretation is beyond my level of statistical expertise.

Answer: Thank you very much for your opinion.

7. Discussion:
Slightly 'wordy'. Could be shortened.

Answer: We agree the Discussion is slightly wordy, and we have shortened. (Page 8, Discussion)

8. Page 9, Para1: Please advise how the figures 65% and 37.26% are arrived at?

Answer: Thank you for this question. 65% is calculated by adding vaccine (0.1550) and health spending (0.4951) in G20 countries in the Dominance analysis column in supplementary table 1. This value is actually 65.01% and has been corrected in the text. Similarly, 37.26% is the sum of global vaccine (0.2708) and health spending (0.1018). (See supplement table 1)

9. Page 11, para 3. The finding that different measures of investment in health resources are correlated with U5MR is interesting. Why do the authors think that GGHE-D is negatively correlated whereas CHE per capita is positively correlated?

Answer: Thanks for this question and it is very interesting. This is actually determined by the coefficients β in Supplementary Table 1. We included U5MR as a dependent variable and health resources as independent

variables in the two-way fixed effect model, so the coefficient β of the independent variable represents the relationship between independent variables and the dependent variable. A positive beta value indicates that for a one-unit increase in the independent variable, the dependent variable also increases by one unit.

10. Page 11, line 15. The model of health care systems (eg private versus public) in relation to equity may also part explain the relationship between health spend and U5MR.

Answer: Your recommendation is highly valuable. The discrepancies in health care system models, particularly the uneven distribution of medical resources, can lead to significant challenges. And we added relevant expression in the text. (Page 9, line 12)

11. Para 6. The authors are right to speculate as to why global vaccination rates have plateaued and / or decreased in recent years. They might mention vaccination hesitancy in this context.

Answer: We sincerely appreciate the insightful suggestion. We agree that vaccination hesitancy is an important factor contributing to the plateauing or decline in global vaccination rates. (Page 10, line 9)

12. Minor:
'What this study adds' section line14: 'have significantly impact U5MR'. Poor English

Answer: Thanks for your careful checks. We are sorry for our carelessness. Follow editor’s advice, we removed the sections ‘What is already known on this topic’, ‘What this study adds’ and ‘How this study might affect research, practice or policy’. Well, the original text should be ‘have a significantly impact on U5MR’.

13. Introduction:
Line 16: repeats sentence 'Domestic general government health expenditure (GGHE-D) as percentage of current health expenditure (CHE)'

Answer: Thank you for pointing this out. We carefully reviewed and searched the original text, but did not find repeated sentences. We added a glossary on health spending indices, which might be helpful.

14. Results:
Page 7 line 27: '..resources'. Might add 'respectively' for clarity.
Page 8 line 2: '..resources'. Might add 'respectively' for clarity.

Answer: Thanks for your advice. We added 'respectively' in the original text. (Page 8, line 4 and 8)

15. Discussion:
Page 11, line 2: ' an indispensable 'what''?

Answer: We sincerely thank you for the careful reading. We made revisions to the original sentence, and the revised version is: *Our findings indicated that vaccination remains an indispensable factor for child health, even in countries with more abundant healthcare resources.* (Page 10, line 1)

Responds to the reviewer’s comments:
Reviewer: 2
Dr. Evans Otieku, University of Ghana, Aarhus University
Comments to the Author:

1. This paper is exciting to read and recognizes the need to dramatically reduce under-five mortality. While making a case for what is known about the study,the authors mentioned 49% reduction in U5MR between 2000-2013,Could they update such information with a more recent estimate of 51%reduction between 2000 and 2022(see Murphy 2024;UNIGCME 2023).

Answer: We sincerely appreciate the valuable suggestion. To ensure the continuity of context in MDG 4, we elected to retain the time spans in the original text without modification. However, we searched the relevant

information as follows: From 2000 to 2023, the number of under-five deaths declined from 10.1 million to 4.8 million, representing a 52% reduction in the mortality rate. (https://data.unicef.org/topic/child-survival/under-five-mortality/)

2. Could they check to see what has been done by the United Nations Inter-Agency Group for Child Mortality Estimate(UNIGCME)to see whether the overarching purpose of this study has not been worked on already.

Answer: Thanks for the advice. The U5MR was 76.7 per 1000 live births in 2000 and 43.7 in 2015, and the data show that the U5MR was reduced by 43% during the Millennium Development Goal (MDG 4) period, the figure that did not meet the expected goal. Extrapolating this trend, the goal of the Sustainable Development Goals (SDGs) remains a significant challenge.

3. It is good to point out that 146 countries are on track to achieve SDG target of 25/1000 by 2030.Should this not inform where to focus the analysis to understand why the remaining countries are lagging behind. Why the specific focus on G20 countries?

Answer: Yes, this is a good question. Firstly, the population of G20 countries approaches two-thirds of the world's total population, and their Gross Domestic Product (GDP) accounts for approximately 85% of the global total. These countries are relatively better at vaccination and health expenditure, and they have the full capability to do so. Indeed, the reality is that the U5MR in G20 countries is significantly lower than the global average. In this study, the contribution of vaccines and health expenditure to reducing the U5MR in both the world and G20 countries is 37.26% and 65.01%, respectively. Based on this, it can be inferred that in other relatively underdeveloped countries, vaccination and health expenditure will play an even more significant role in reducing the U5MR.

4. Could they expand a bit more about how they addressed heterogeneity in this comparative analysis?

Answer: Thanks for your question. The primary strength of the two-way fixed effects model is its concurrent control of two distinct forms of heterogeneity. Firstly, individual fixed effects pertain to characteristics that are unique to each country and remain constant over time, such as geographical conditions. Secondly, time fixed effects represent temporal shocks that are uniformly experienced by all countries, for instance, the global financial crisis.

5. Could they expand the reasons for the log-linear model.

Answer: Thanks for your valuable question. Mortality rate data often demonstrates an exponential change trend, a non-linear characteristic that cannot be accurately captured by the ordinary linear model. However, employing logarithmic transformation can convert this exponential relationship into a linear one, thereby enabling the identification of trend turning points. Furthermore, logarithmic transformation can stabilize variance, thus preventing the fluctuation of data in high mortality rate areas from overshadowing crucial turning signals.

6. A better description of the statistical analysis will help, especially with regards to justification and all the underlying assumptions considered in model development.

Answer: Thanks for your suggestion. The variables with Variance Inflation Factor (VIF)>10 were excluded by the multicollinearity test. The decision between using the fixed effect model or the random effect model was assessed through the Hausman test. Whether to add time fixed effect was determined by the joint significance test. (Page 4, Statistical analysis)

VERSION 2 - REVIEW

**Reviewer**	**2**
**Name**	**Otieku, Evans**

Affiliation **University of Ghana, Institute of Statistical, Social and Economic Research**

Date **17-Sep-2025**

COI

The revised manuscript is satisfactory, and I recommend its acceptance for publication.

VERSION 2 - AUTHOR RESPONSE

Reviewer: 2
Dr. Evans Otieku, University of Ghana, Aarhus University
Comments to the Author:

1. The revised manuscript is satisfactory, and I recommend its acceptance for publication.

Answer: Thank you for sharing the positive outcome. Please accept our deepest gratitude for the guidance, and your insights have been instrumental in improving the quality of our work.
